# The effect of breed and body weight at slaughter on histochemical muscle fiber characteristics and meat quality of longissimus lumborum and semitendinosus lamb muscles

**DOI:** 10.5194/aab-66-439-2023

**Published:** 2023-12-13

**Authors:** Witold Rant, Aurelia Radzik-Rant

**Affiliations:** Institute of Animal Sciences, Warsaw University of Life Sciences-SGGW, Ciszewskiego St. 8, 02-786 Warsaw, Poland

## Abstract

The skeletal muscles of mammals are composed of fibers of different morphological, metabolic and functional characteristics. The properties of muscle fibers may be determined genetically as well as by environmental factors such as the age of the animals, their physical activity, the level of nutrition, or the selection intensity. The present study was conducted to determine the influence of genotype (Polish Lowland vs. Polish Heath) and body weight at slaughter (23–25 kg vs. 35–40 kg) of lambs on histological characteristics of muscle fibers in musculus longissimus lumborum (LL) and musculus semitendinosus (ST) skeletal muscles and their impact on chosen meat quality features. Three types of muscle fibers were identified: slow-twitch oxidative (STO), fast-twitch oxidative (FTO) and fast-twitch glycolytic (FTG). Differences in the diameters of individual fiber types between the LL and ST muscles have been found in both investigated genotypes. The diameters of the analyzed types of fibers were usually larger in the ST muscle compared to the LL muscle. The lambs of the more primitive Polish Heath breed were characterized by a smaller diameter of all fiber types, especially in the LL muscle. The higher proportion of STO fibers and the lower proportion of FTO fibers have been found in the LL muscle of Polish Heath lambs in the group with lower body weight. The breed of lambs, muscle type and slaughter body weight had an impact on some meat quality characteristics, especially color, intramuscular fat content and expressed juice.

## 1 Introduction

The skeletal muscles of mammals are composed of fibers of different morphological, metabolic and functional characteristics. The properties of muscle fibers can be determined genetically as well as by environmental factors such as the age of the animals, their physical activity, the level of nutrition or the selection intensity (Rehfeldt et al., 2000; Picard et al., 2006; Wojtysiak et al., 2010; De Marzo et al., 2012; Reimers et al., 2014).

The muscle fibers vary depending on their metabolic properties and speed of contraction and are divided into three main types: slow-twitch oxidative (STO) or type I or β -red ( β R); fast-twitch oxidative (FTO) or type IIA or α -red ( α R); and fast-twitch glycolytic (FTG) or type IIB or α -white ( α W) (Canepari et al., 2010; Schiaffino and Reggiani, 2011; Furuichi et al., 2014).

Muscle contraction requires energy from adenosine triphosphate (ATP). In STO fibers the oxidative pathway is used for ATP regeneration. These fibers are rich in mitochondria and in myoglobin, which is the oxygen carrier. They are also characterized by higher concentrations of intracellular lipids and low contents of glycogen and glucose (Choi and Kim, 2009). In contrast, the FTG fibers use the glycolytic pathway, contain fewer mitochondria and are nearly devoid of myoglobin. Compared to STO fibers, they contract 3 times faster but exhibit low resistance to fatigue (Picard et al., 2002). In turn, FTO fibers can use the energy from anaerobic pathways where pyruvate is converted into lactic acid in the sarcoplasm as well as from aerobic pathways through which pyruvate is oxidized by the mitochondria. They contract more slowly than FTG fibers (Schiaffino and Reggiani, 2011).

The various types of fibers are present in all skeletal muscles but in different proportions. These proportions may affect the metabolic properties of muscles, their characteristics and the meat quality of livestock animals (Peinando et al., 2004; Velotto et al., 2005; Lee et al., 2010; Kim et al., 2013; Wojtysiak and Połtowicz, 2014; Listrat et al., 2016).

According to Choi et al. (2006), 80 %–90 % of FTO fibers and 5 %–10 % of STO fibers were recorded in pig longissimus dorsi muscle, while in musculus vastus intermedius the share of oxidative fibers was 70 %–80 %. In beef biceps brachii muscle higher proportions of STO fibers have been found compared to longissimus dorsi and biceps femoris muscles (Krichofer et al., 2002). Sazili et al. (2005), studying the distribution of fiber types in various sheep muscles, found a significant advantage of fast-twitch fibers (FTO + FTG) over STO fibers in longissimus dorsi muscle, while in semitendinosus muscle these proportions were 83.2 % and 16.8 %, respectively, and in the musculus trapezius they were 45.5 % and 54.5 %, respectively. The abovementioned results confirm that the proportion of fiber types depends on the specific physiological function of the muscle. Muscles more resistant to fatigue such as postural and respiratory muscles are characterized by a higher content of slow-twitch oxidative fibers, while muscles responsible for movement show a predominance of fast-twitch glycolytic fibers (Briand et al., 1981).

The proportion of muscle fibers in the same muscle may vary depending on the animal feeding system. In studies conducted on goat kids, it was found that those reared under the milk replacer method showed a lower percentage of slow oxidative fibers and a higher percentage of fast type fibers in comparison to animals reared under the natural suckling method (Rivero et al., 2022). In turn, in the research by Hou et al. (2020), a significantly higher share of slow-twitch oxidative fibers in the biceps femoris muscle was found in sheep kept on pasture compared to the group kept in the pen and fed a controlled diet.

The composition of muscle fibers is also influenced by breed or animal genotype as well as by changes in their structure during the growth and development of animals (Solomon et al., 1981; Wegner et al., 2000; Kłosowska and Fiedler, 2003; Greenwood et al., 2007; Velotto et al., 2010; Siqin et al., 2017; Sirin et al., 2017).

These changes may have an impact on the meat quality and its acceptance by the consumer perceived by physicochemical features such as color, juiciness, tenderness or amount of intramuscular fat. The high myoglobin content in muscles with a larger proportion of slow-twitch oxidative fibers results in a higher red color intensity, while a high proportion of glycolytic fibers results in the production of white meat (Listrat et al., 2016). An increased proportion of slow-twitch oxidative fibers is associated with improved meat juiciness because the proteins in these muscles have a better ability to bind water (Ryu and Kim, 2006). Tenderness is a key indicator for evaluating meat quality, which is an important factor affecting consumer and market acceptance. In meat the improvement in tenderness may be associated with the increase in oxidative fiber proportion and higher intramuscular fat content in these muscles (Wojtysiak et al., 2010; Listrat et al., 2016).

The purpose of the current study was to determine the influence of genotype and body weight of lambs on histological characteristics of muscle fibers in musculus longissimus lumborum (LL) and musculus semitendinosus (ST) skeletal muscles and their impact on selected traits of meat quality.

## 2 Materials and methods

The experiment was conducted on 20 Polish Wrzosówka (PW) ram lambs and 20 ram lambs of Polish Lowland (PL) sheep. Polish Wrzosówka is an indigenous breed characterized by a thin, small and proportionally built figure, while Polish Lowland is a typical meat-and-wool-purpose sheep.

All the ram lambs came from twin litters and were nursed by ewes to the age of 100 d. During the suckling period, from the second week of life, the lambs, in addition to their mother's milk, received crushed oats and meadow hay. After weaning the animals were kept in a barn on straw bedding under uniform environmental conditions with constant zootechnical and veterinary supervision.

The lambs were fed in a group according to the standards for fattening lambs (Osikowski et al., 1998). The diet of the lambs was based on meadow hay and concentrate containing 30 % oat meal, 40 % barley meal, 19 % wheat bran, 10 % rapeseed meal and 1 % mineral mixture. The animals were fed twice a day, and the dose level was adjusted to the requirements. The animals had constant access to water. The lambs were slaughtered in two weight standards: 23–25 kg (10 animals for each breed group) and 35–40 kg (10 animals for each breed group). Before slaughter, ram lambs were fasted for 12 h and weighed. Then, they were taken to an abattoir and slaughtered according to Council Regulation (EC) No. 1099/2009 of 24 September 2009 (Acts Office EU dated 18 November 2009 L 303/1).

The meat samples for histochemical analysis were taken from the right side of the carcass within 45 min post mortem from the middle of the lumbar spine of the LL muscle between the fourth and fifth lumbar vertebrae and from the middle of the ST muscle. The samples were cut longitudinally to the muscle fibers into 0.5 × 0.5 × 0.8 cm blocks and frozen in liquid nitrogen, where they were stored until further analysis.

Then the LL and ST muscles from the right and left sides of each carcass were dissected, trimmed of visible connective and adipose tissues, vacuum-packed and transported in the refrigerator to the laboratory to perform analysis of meat quality. Samples from the right side of a carcass were analyzed to determine pH, intramuscular fat (IMF), total collagen content and expressed juice. The meat color and shear force were tested on samples from the left side.

Frozen samples were cut into 10 µ m thick transverse serial sections using a cryostat microtome at -20 ${}^{\circ }$ C. The three sections for each muscle were placed on a glass slide and were subjected to staining using combined histochemical reactions: nicotinamide adenine dinucleotide dehydrogenase (NADH)-tetrazolium reductase and the acid-preincubated myofibrillar ATPase at pH 4.2 according to the method described by Horak (1983). It allowed simultaneous evaluation of the ATPase activity and oxidative capacity of the muscle fibers. This combined staining method allowed us to distinguish between three muscle fiber types: fibers stained dark are classified as STO, intermediate as FTO and light as FTG. For each muscle or section, the three randomly selected areas were chosen and the number of fibers of each type within the known area were counted. The measurements of individual muscle fiber diameters were made on approximately 300 fibers for each muscle using the MultiScan 14.02 image analysis system. To minimize any errors associated with fibers that may not have been cut at right angles or that were irregular, diameters were taken as the smallest diameter across the fiber (Dubowitz et al., 1973). The percentages of STO, FTO and FTG fibers refer to the ratio of the number of fibers counted for each fiber type to the total number of counted fibers.

The pH of the meat was measured after 24 h post mortem using an Elmetron CP-411 pH meter with a dagger electrode calibrated at pH values of 4.0, 7.0 and 9.0.

To determine the expressed juice, 0.3 g of minced meat was placed on Whatman filter paper no. 1 and held at a pressure of 2 kg for 5 min. The outline area of the expressible juice and the meat film was traced, and two areas were measured using a planimeter (Grau and Hamm, 1953). The results have been calculated in meat (cm ${}^{2}$ g ${}^{-1}$ ).

Total collagen and IMF content were evaluated using the near-infrared (NIR) transmission method (PN-A-82109). The samples were homogenized in an Elektrolux DITO K35 processor. Then, samples were placed in a measuring cell of a FoodScan analyzer. The device uses the NIR transmission method within the 850–1050 nm range and is fitted with artificial neural network (ANN) calibration developed using a model of ANNs. The analysis is performed by indicating in the computer program the number of 16 measurements in the sample, and then the program automatically calculates the average and presents the result.

The color LL and ST samples were measured on the meat surface, after 30 min of blooming, using a Minolta CR-410 (Konica Minolta) colorimeter. The lightness ( $L^{*}$ ), redness ( $a^{*}$ ) and yellowness ( $b^{*}$ ) were recorded three times at three different locations. Color saturation ( $C^{*}$ ) and hue angle ( $H^{*}$ ) were calculated according to AMSA guidelines (AMSA, 2012).

To evaluate the shear force value, the samples of LL and ST muscles were wrapped in baking paper and heated in a convection oven at 90 ${}^{\circ }$ C until they reached the endpoint temperature (70 ${}^{\circ }$ C) in the approximate geometric center of the sample. Then, the samples were cooled and kept overnight in a chiller at 4 ${}^{\circ }$ C. Three cores (1.25 cm in diameter) parallel to the muscle fiber orientation were cut from each sample. The shear force was determined as the maximum force ( N ) perpendicular to the fibers using ZWIKI Roell type Z 2.5 equipped with a Warner–Bratzler blade. The average value from three replications for each sample was used for statistical analysis.

A statistical analysis of the data was performed using the SPSS 23.0 packet software (SPSS Base 23.0, 2016). The data distribution was verified using a Shapiro–Wilk test. As the distribution was normal, a linear model that included the effects of breed, body weight at slaughter and interaction breed × body weight at slaughter was used. All the effects were tested against residual middle squares to determine the level of significance. P values < 0.05 were considered statistically significant. The results are presented as the least-squares means (LSMs) for each trait and standard error (SE).

## 3 Results and discussion

The analysis of the histological profile of the muscle fibers of Polish Lowland ram lambs slaughtered at body weights of 23–25 and 35–40 kg showed that animals from a higher weight category were characterized by larger ( P<0.05 ) diameters of all the identified muscle fibers STO, FTO and FTG in the LL muscle. However, there were no differences in the muscle fiber diameter between the weight categories in the ST muscle (Table 1). The mean diameter of STO fibers was greater ( P<0.05 ) in the LL muscle compared to the ST muscle in PL rams slaughtered at a body weight of 35–40 kg. In turn, the diameters of FTG fibers were smaller ( P<0.05 ) in the LL muscle within both investigated weight categories (Table 1). The analysis of meat quality characteristics showed that the ST muscle was characterized by less expressed juice compared to the LL muscle. The lower value ( P<0.05 ) of this parameter was found within both weight categories. The body weight at slaughter has an effect on the changes in meat color. Both LL and ST muscles in lambs slaughtered at the higher body weight showed an increase ( P<0.05 ) in the value of redness ( $a^{*}$ ). The greater saturation of redness was confirmed by an increase in the $C^{*}$ value ( P<0.05 ) and a decline in $H^{*}$ . Investigated muscles from the group slaughtered at 35–40 kg were also characterized by a higher ( P<0.05 ) content of intramuscular fat (Table 1).

**Table 1:** The mean values of fiber diameter ( µ m), fiber proportion (%) and meat quality traits of musculus longissimus lumborum (LL) and musculus semitendinosus (ST) muscles in Polish Lowland ram lambs slaughtered at 23–25 and 35–40 kg body weight.

Trait	BW 23–25 kg		BW 35–40 kg		SE	Effect		
	LL	ST	LL	ST		M	BWE	M × BW
STO ( µ m)	25.17 ${}^{x}$	27.20	29.97 ${}^{\mathrm{ay}}$	26.30 ${}^{b}$	0.99	${}^{*}$	${}^{*}$	${}^{*}$
FTO ( µ m)	22.75 ${}^{x}$	24.73	27.04 ${}^{y}$	25.88	0.70	ns	${}^{*}$	${}^{*}$
FTG ( µ m)	25.26 ${}^{\mathrm{ax}}$	31.11 ${}^{b}$	28.47 ${}^{\mathrm{ay}}$	32.27 ${}^{b}$	0.83	${}^{*}$	${}^{*}$	${}^{*}$
STO (%)	12.88	12.61	14.06	13.81	1.01	ns	ns	ns
FTO (%)	45.04	43.61	48.26 ${}^{a}$	43.60 ${}^{b}$	1.14	${}^{*}$	ns	ns
FTG (%)	42.08	43.79	37.68 ${}^{a}$	42.59 ${}^{b}$	1.08	${}^{*}$	ns	ns
pH24	5.98	6.08	5.81	5.91	0.12	ns	ns	ns
$L^{*}$ (lightness)	42.46	44.72	39.34	41.43	1.32	ns	ns	ns
$a^{*}$ (redness)	14.23 ${}^{x}$	13.83 ${}^{x}$	16.75 ${}^{y}$	16.28 ${}^{y}$	0.63	ns	${}^{*}$	ns
$b^{*}$ (yellowness)	4.71	5.18	4.03	4.44	0.49	ns	ns	ns
$C^{*}$ (chroma)	15.00 ${}^{x}$	14.79 ${}^{x}$	17.31 ${}^{y}$	16.97 ${}^{y}$	0.66	ns	${}^{*}$	ns
$H^{*}$ (hue angle)	18.30 ${}^{x}$	20.53 ${}^{x}$	13.37 ${}^{y}$	15.03 ${}^{y}$	1.54	ns	${}^{*}$	ns
IMF (%) (intramuscular fat)	3.12 ${}^{x}$	3.28 ${}^{x}$	4.60 ${}^{y}$	4.76 ${}^{y}$	0.38	ns	${}^{*}$	ns
Total collagen (%)	0.77	0.98	0.70	0.89	0.07	ns	ns	ns
Expressed juice (cm ${}^{2}$ g ${}^{-1}$ )	27.03 ${}^{a}$	22.51 ${}^{b}$	27.19 ${}^{a}$	22.64 ${}^{b}$	1.07	${}^{*}$	ns	ns
Shear force ( N )	46.07	50.57	46.61	51.16	5.41	ns	ns	ns

BW – body weight; M – muscle effect; BWE – body weight effect; M × BW – interaction of muscle and body weight; SE – standard error. STO – slow-twitch oxidative fibers; FTO – fast-twitch oxidative fibers; FTG – fast-twitch glycolytic fibers. Different superscripts in the same row represent significant differences between individual muscles (within body weights) – ${}^{a,b}$ P<0.05 . Different superscripts in the same row represent significant differences between body weight (within individual muscles) – ${}^{x,y}$ P<0.05 ; ${}^{*}$ P<0.05 ; ns – not significant.

**Table 2:** The mean values of fiber diameter ( µ m), fiber proportion (%) and meat quality traits of LL and ST muscles in Polish Wrzosówka ram lambs slaughtered at 23–25 and 35–40 kg of body weight.

Trait	BW 23–25 kg		BW 35–40 kg		SE	Effect		
	LL	ST	LL	ST		M	BWE	M × BW
STO ( µ m)	21.10 ${}^{a}$	26.13 ${}^{b}$	24.71	26.03	1.13	${}^{*}$	ns	ns
FTO ( µ m)	21.70 ${}^{a}$	25.94 ${}^{b}$	22.25 ${}^{a}$	26.45 ${}^{b}$	0.93	${}^{*}$	ns	ns
FTG ( µ m)	24.88 ${}^{a}$	30.12 ${}^{\mathrm{bx}}$	25.95 ${}^{a}$	32.51 ${}^{\mathrm{by}}$	0.99	${}^{*}$	${}^{*}$	ns
STO (%)	16.73	15.71 ${}^{x}$	17.13	18.58 ${}^{y}$	0.69	ns	${}^{*}$	ns
FTO (%)	40.20	40.69	41.33	40.48	0.91	ns	ns	ns
FTG (%)	43.07	43.60	41.54	40.94	1.03	ns	ns	ns
pH24	5.98	6.086	5.83	5.931	0.16	ns	ns	ns
$L^{*}$ (lightness)	36.57	38.51 ${}^{x}$	34.96	35.77 ${}^{y}$	0.99	ns	${}^{*}$	ns
$a^{*}$ (redness)	17.52 ${}^{x}$	17.03 ${}^{x}$	20.22 ${}^{y}$	19.66 ${}^{y}$	0.39	ns	${}^{*}$	ns
$b^{*}$ (yellowness)	4.04	4.45	3.93	4.33	0.49	ns	ns	ns
$C^{*}$ (chroma)	18.05 ${}^{x}$	17.69 ${}^{x}$	20.62 ${}^{y}$	20.16 ${}^{y}$	0.43	ns	${}^{*}$	ns
$H^{*}$ (hue angle)	12.80	14.41	10.95	12.36	1.39	ns	ns	ns
IMF (%) (intramuscular fat)	1.81	1.95	2.37	2.51	0.33	ns	ns	ns
Total collagen (%)	0.66 ${}^{\mathrm{ax}}$	0.83 ${}^{\mathrm{bx}}$	0.82 ${}^{y}$	1.03 ${}^{y}$	0.03	${}^{*}$	${}^{*}$	${}^{*}$
Expressed juice (cm ${}^{2}$ g ${}^{-1}$ )	21.93	18.26	23.73 ${}^{a}$	19.76 ${}^{b}$	1.36	${}^{*}$	ns	ns
Shear force ( N )	40.19	44.11	46.38	50.90	5.80	ns	ns	ns

There were no statistically significant differences in the diameters of the STO and FTO fibers in the longissimus lumborum and semitendinosus muscles in the Polish Wrzosówka rams slaughtered at different body weights. Only FTG fibers were characterized by a larger diameter ( P<0.05 ) in the ST muscle in ram lambs slaughtered at higher body weights (Table 2).

In contrast, the differences in the muscle fiber diameters between LL and ST muscles were evident regardless of the slaughter body weight, except for STO fibers in the higher weight category. The diameters of the FTO and FTG fibers were larger ( P<0.05 ) in the ST muscle compared to the LL muscle (Table 2). The ST muscle was also characterized by a higher collagen content, which was statistically confirmed ( P<0.05 ) in lambs slaughtered at a body weight of 23–25 kg. Similarly to the group of PL lambs, the ST muscle was characterized by a lower value of expressed juice, especially in the group slaughtered in the higher weight category ( P<0.05 ). The decline ( P<0.05 ) of lightness ( $L^{*}$ ) was recorded in the ST muscle of PW lambs slaughtered at a body weight of 35–40 kg. In this group there was also an increase ( P<0.05 ) in the values of redness ( $a^{*}$ ), chroma ( $C^{*}$ ) and total collagen content in both tested muscles compared to lambs from the lower weight category (Table 2).

The results obtained by other authors confirm the increase in muscle fiber diameter in animals slaughtered at a higher body weight in both sheep (Peinando et al., 2004; Wojtysiak et al., 2010) and cattle (Jurie et al., 2005; Młynek et al., 2006).

The influence of age, and thus higher body weight, on muscle fiber diameter increase was also observed by Siqin et al. (2017) in longissimus dorsi, biceps femoris and triceps brachii muscles of Wuzhumuqin sheep. Velotto et al. (2010), examining the muscle fiber profiles in the longissimus dorsi and semitendinosus muscles in lambs of the Laticauda breed slaughtered at 60 and 120 d of age, also confirmed the greater diameters of all types of muscle fibers in older lambs. In contrast, unlike in the present study, the authors did not show differences in the fiber diameters between investigated muscles at the same slaughter age.

Analyzing the diameters of various types of muscle fibers, it can be noticed that the FTG fibers were characterized by the largest value of this feature in both the lower and higher slaughter weight categories in both studied genotypes. In contrast, in the LL muscle, the STO fibers were slightly larger in diameter than the FTG fibers of the heavier lambs of the PL breed (Tables 1 and 2). This is consistent with the finding of Suzuki and Cassens (1983) that in sheep the sizes of slow-twitch oxidative and fast-twitch glycolytic fibers are similar, and the FTG fibers are not always larger than the STO fibers. There were no statistically significant differences in the percentages of individual muscle fibers between the weight categories, except for a greater ( P<0.05 ) content of STO fibers in the ST muscle of heavier PW lambs (Tables 1 and 2). It was also observed that, in PL ram lambs slaughtered at a higher body weight, the percentage of STO fibers in the LL muscle was 3.4 % higher compared to lambs from the lower weight category.

In the LL muscle of Polish Lowland and Polish Wrzosówka lambs slaughtered at a higher body weight, the content of FTO fibers increased, although not statistically significantly, by about 6.7 % and 2.7 %, respectively, while FTG fibers decreased by 11.6 % and 3.6 %, respectively. Similar but much smaller differences in the share of FTO and FTG fibers between weight categories were observed for the ST muscle (Tables 1 and 2).

The comparison of the examined muscles within weight categories only showed a higher ( P<0.05 ) content of FTO fibers and a lower content ( P<0.05 ) of FTG fibers in the longissimus lumborum muscle compared to the semitendinosus muscle in PL lambs from the higher body weight category (Table 1). In PW ram lambs, slaughtered at both lower and higher body weights, no statistically significant differences were found in the percentage of individual fiber types between muscles (Table 2).

Studies by many authors indicate a lack of clear changes in the proportions of muscle fibers depending on body weight at slaughter. Solomon et al. (1980) did not find differences in the percentage of fibers between higher and lower body weights at slaughter in lambs. Wojtysiak et al. (2010) also found no statistically significant differences in the content of individual muscle fibers in the longissimus lumborum muscle of lambs slaughtered at different weight standards. However, as in the presented study, the share of STO and FTO fibers increased and that of FTG fibers decreased in animals with a higher body weight. The increase in the proportion of slow-twitch oxidative fibers and the decrease in fast-twitch fibers (FTO + FTG) in animals with a higher body weight was also noted by Therkildsen et al. (2002), Peinando et al. (2004) and Młynek et al. (2006). In turn, Moody et al. (1980) found that, with increasing body weight at slaughter, the proportion of STO fibers decreased and those of FTO and FTG fibers increased.

Compared to the present study, Greenwood et al. (2007), analyzing the muscle fibers in lambs of two breeds of sheep at ages of 4, 8, 14 and 22 months, showed greater differences in shares of STO and FTO fibers between the examined LD (longissimus dorsi) and ST muscles. Similarly, Siqin et al. (2017), in sheep of the local Wuzhumuqin breed, examining the development of muscle fibers in longissimus dorsi, biceps femoris and triceps brachii from 1 to 18 months of age, showed a differentiation in their proportions depending on the examined muscle in all the studied periods.

In the present study the body weight at slaughter influenced the same meat color coordinates in both studied breeds. The LL and ST muscles were characterized by lower values of lightness ( $L^{*}$ ) and higher redness ( $a^{*}$ ) in lambs slaughtered at a higher body weight. These results are consistent with those given by Preziuso and Russo (2004) for longissimus thoracis, semitendinosus and triceps brachii muscles from Chianina beef cattle slaughtered at two different ages. The differences observed in meat color may be due to different muscle fiber compositions and myoglobin contents in which the concentration is higher in muscles with more slow oxidative fibers (Picard et al., 2002; Mancini and Hunt, 2005). In the present study, the lambs slaughtered at a body weight at 35–40 kg showed a higher proportion of STO fibers in both muscles, which can explain the differences in the parameters determining the color of the meat (Tables 1 and 2).

There were no statistical differences in the intramuscular fat content between slaughter categories in the PW group, while differences were noted in the PL lambs. In this group, higher body weight resulted in higher fat content in both LL and ST muscles. Martinez-Cerezo et al. (2005) also reported an increase in fat content in the meat of lambs slaughtered at a higher body weight. According to Prache et al. (2022), muscle tissue grows steadily after birth at the same pace as the frame, whereas fat deposits are slower to develop. This means, as the animal grows towards maturity and increases in body weight, that the proportion of fat in the muscle increases.

In the present study differences in the total collagen content were found between both muscle types and body weight at slaughter in PW lambs. ST muscle was characterized by a higher content of this component compared to LL muscle, especially in the group of the lower weight category ( P<0.05 ). Considering the effect of muscle type on the collagen content, Tschirhart-Hoelscher et al. (2006) found a higher content of it in gluteus medius muscle than in longissimus dorsi muscle. Also, He et al. (2023) confirmed in their study a higher proportion of collagen in the semitendinosus muscle compared to the longissimus dorsi muscle in Wuzhumuqin sheep. Similarly to the present study, the authors also confirmed an increase in the collagen content in the examined muscles in older animals slaughtered at higher body weights.

Analyzing the expressed juice, it was confirmed that, within both investigated breeds and slaughter weight categories, the ST muscles showed a better ability to hold their own water in comparison to the LL muscles (Tables 1 and 2). According to Huff-Lonergan and Lonergan (2005), this parameter is determined by many interacting factors, e.g., the muscle structure, pH or treatment of meat after slaughter. Therefore, the differences between muscles in the present study could to some extent be determined by a slightly higher pH in the ST muscle as well as its better strength of proteins in binding water.

The histological profiles of the muscle fibers of PL and PW ram lambs slaughtered at body weight 23–25 kg are presented in Table 3.

**Table 3:** The mean values of fiber diameter ( µ m), fiber proportion (%) and meat quality traits of LL and ST muscles in Polish Heath and Polish Lowland ram lambs slaughtered at 23–25 kg body weight (bold font indicates statistically significant differences).

Trait	LL				ST			
	PW	PL	SE	P value	PW	PL	SE	P value
STO ( µ m)	21.10	25.17	1.15	**0.028**	26.13	27.20	0.89	0.428
FTO ( µ m)	21.70	22.75	0.86	0.421	25.94	24.73	0.76	0.295
FTG ( µ m)	24.88	25.26	0.91	0.784	30.12	31.11	0.87	0.453
STO (%)	16.73	12.88	0.95	**0.013**	15.71	12.61	1.21	0.083
FTO (%)	40.20	45.04	1.13	**0.009**	40.69	43.61	1.05	0.060
FTG (%)	43.07	42.08	1.22	0.589	43.60	43.79	0.88	0.898
pH24	5.98	5.98	0.16	0.985	6.09	6.08	0.16	0.985
$L^{*}$ (lightness)	36.57	42.46	1.09	**0.002**	38.51	44.72	1.15	**0.002**
$a^{*}$ (redness)	17.52	14.23	0.40	**0.001**	17.03	13.83	0.39	**0.001**
$b^{*}$ (yellowness)	4.04	4.71	0.47	0.334	4.45	5.18	0.52	0.334
$C^{*}$ (chroma)	18.05	15.00	0.45	**0.001**	17.69	14.79	0.45	**0.001**
$H^{*}$ (hue angle)	12.80	18.29	1.39	**0.013**	14.41	20.53	1.54	**0.013**
IMF (%) (intramuscular fat)	1.81	3.12	0.30	**0.007**	1.95	3.27	0.30	**0.007**
Total collagen (%)	0.66	0.77	0.05	0.120	0.83	0.98	0.06	0.120
Expressed juice (cm ${}^{2}$ g ${}^{-1}$ )	21.93	27.03	1.47	**0.026**	18.26	22.51	1.23	**0.026**
Shear force ( N )	40.19	46.07	5.80	0.484	44.11	50.57	6.37	0.484

PW – Polish Wrzosówka ram lambs; PL – Polish Lowland ram lambs; SE – standard error. STO – slow-twitch oxidative fibers; FTO – fast-twitch oxidative fibers; FTG – fast-twitch glycolytic fibers.

The diameters of the STO fibers were larger ( P<0.05 ) only in the LL muscle of the PL lambs compared to the PW lambs. There were no statistically significant differences in the diameters of other types of muscle fibers between genotypes in the LL muscle as well as between STO, FTO and FTG fibers in the semitendinosus muscle. It should be noted that Polish Wrzosówka ram lambs, despite the lack of statistical confirmation, showed smaller diameters of all types of fibers, especially in the longissimus lumborum muscle. Similarly, in animals slaughtered at higher body weights, the LL muscle of the PW lambs was characterized by lower ( P<0.05 ) diameters of STO, FTO and FTG fibers compared to the PL ram lambs. More equal values of this parameter between the studied genotypes were recorded in the ST muscle (Table 4).

A comparison of the quality parameters of the LL and ST muscles between the PL and PW, slaughtered in both weight categories, showed that the muscles of the PW lambs were darker compared to the PL lambs (the lower value of the $L^{*}$ parameter). Their muscles were also characterized by a greater saturation of redness, which was confirmed by higher ( P<0.05 ) values of $a^{*}$ and $C^{*}$ parameters (Tables 3 and 4). Both muscles of PW lambs also showed a lower ( P<0.05 ) content of intramuscular fat as well as better values of expressed juice (Tables 3 and 4).

**Table 4:** The mean values of fiber diameter ( µ m), fiber proportion (%) and meat quality traits of LL and ST muscles in Polish Wrzosówka and Polish Lowland ram lambs slaughtered at 35–40 kg body weight (bold font indicates statistically significant differences).

Trait	LL				ST			
	PW	PL	SE	P value	PW	PL	SE	P value
STO ( µ m)	24.71	29.97	1.24	**0.004**	26.03	26.30	0.84	0.815
FTO ( µ m)	22.25	27.04	0.64	**0.001**	26.45	25.88	0.60	0.491
FTG ( µ m)	25.95	28.47	0.58	**0.003**	32.51	32.27	0.43	0.688
STO (%)	17.13	14.06	0.77	**0.007**	18.58	13.81	0.58	**0.001**
FTO (%)	41.33	48.26	1.69	**0.006**	40.48	43.60	0.84	**0.009**
FTG (%)	41.54	37.68	1.78	0.124	40.94	42.59	0.78	0.122
pH24	5.83	5.81	0.11	0.901	5.93	5.91	0.11	0.901
$L^{*}$ (lightness)	34.96	39.34	1.17	**0.016**	35.77	41.43	1.22	**0.004**
$a^{*}$ (redness)	20.22	16.75	0.59	**0.001**	19.66	16.28	0.57	**0.001**
$b^{*}$ (yellowness)	3.93	4.03	0.48	0.883	4.33	4.44	0.53	0.883
$C^{*}$ (chroma)	20.62	17.31	0.61	**0.001**	20.16	16.97	0.60	**0.002**
$H^{*}$ (hue angle)	10.95	13.37	1.46	0.258	12.36	15.03	1.63	0.261
IMF (%) (intramuscular fat)	2.37	4.60	0.39	**0.001**	2.51	4.76	0.39	**0.001**
Total collagen (%)	0.82	0.70	0.05	0.163	1.03	0.89	0.07	0.163
Expressed juice (cm ${}^{2}$ g ${}^{-1}$ )	23.73	27.19	0.90	**0.014**	19.76	22.64	0.75	**0.014**
Shear force ( N )	46.38	46.61	5.42	0.976	50.90	51.16	5.95	0.976

Comparisons between different breeds of animals, and above all between wild and domestic animals, suggest that selection to improve growth rate and meat performance resulted in an increase in muscle fiber diameter (Ashmore et al., 1972). This has been confirmed in the present study, because Polish Wrzosówka sheep, as a more primitive breed not selected for meat performance, was characterized by a smaller diameter of muscle fibers compared to the meat-and-wool-purpose PL lambs, which was especially visible in the LL muscle of animals slaughtered at a higher body weight. In turn, Fantová et al. (2015) found significantly thicker (34.05 µ m vs. 21.10 µ m) STO fibers and, to a lesser extent, thicker FTO and FTG fibers in the longissimus lumborum muscle of German Heath lambs slaughtered at 150 d of age compared to PW rams from the present study. It should be noted that the size of muscle fibers, apart from genotype or age, may be influenced by other factors such as maintenance and nutrition conditions (Greenwood et al., 2007).

Analyzing the proportions of fiber types in the investigated genotypes, a greater ( P<0.05 ) share of STO fibers and a lower ( P<0.05 ) percentage of FTO fibers in the LL muscle of PW slaughtered in the 23–25 kg weight category have been noted (Table 3). The similar results were observed for the ST muscle in the group of lambs slaughtered at body weight 35–40 kg (Table 4). The share of FTG fibers did not differ significantly between the genotypes in both weight categories.

The higher proportion of STO fibers and the lower percentage of FTO fibers in the LL muscle of PW observed in the conducted study are consistent with the results obtained by Solomon et al. (1981) for genotypes with the share of Finnish sheep belonging to the same group of northern European short-tailed sheep as the Polish Wrzosówka. In turn, Borys et al. (2005) compared the Finnish breed with meat sheep (Île-de-France and Suffolk) and noted a higher proportion of STO fibers in meat breeds, while the longissimus lumborum muscle of Finnish sheep contained more FTO and FTG fibers. In other studies, the meat-purpose Texel breed was characterized by a lower proportion of STO fibers and a higher content of fast-twitch fibers (FTO + FTG) compared to the multi-purpose Scottish Blackface breed (Bunger et al., 2009). Similarly, more oxidative fibers were found in the LD and ST muscles in Merino sheep compared to the Poll Dorset meat breed (Greenwood et al., 2007).

The differences in the proportions of muscle fibers between the six Turkish breeds were noted by Sirin et al. (2017). The number of FTG fibers in the LD muscle of Markaraman sheep was greater than in the other studied breeds. On the other hand, Awassi lambs had a greater number of FTO fibers in the semitendinosus muscle. The abovementioned studies and research carried out on other animal species confirm the influence of the genotype on the histological profile of muscle fibers, indicating that the muscles of multipurpose breeds are characterized by a higher proportion of oxidative fibers with a smaller diameter, while a higher content of glycolytic fibers is characteristic in animals selected for meat performance (Picard et al., 2006; Ryu et al., 2008).

The lower value of the $L^{*}$ parameter in PW rams slaughtered at body weights of 23–25 and 35–40 kg indicates that this breed has a darker color of meat (Tables 3 and 4). The meat of these animals was also characterized by a more intense red shade. This suggests that the PW, as a more primitive breed with a higher content of oxidative muscle fibers, contains a higher concentration of myoglobin in the meat. Scottish Blackface sheep also had a darker meat color compared to the Suffolk and Texel breeds (Carson et al., 2001). On the other hand, Grześkowiak et al. (2003) did not find statistically significant differences between the studied genotypes of lambs, although the lowest value of the $L^{*}$ parameter was found in the meat of Finnish lambs.

The examined genotypes differed in terms of intramuscular fat content regardless of weight category (Tables 3 and 4). The lower content of IMF in the meat of the PW breed was confirmed in a previous study by Radzik-Rant et al. (2014). Intramuscular fat affects the culinary value of meat. The recommended content of this component according to Hopkins et al. (2006) in sheep meat should be within 3 %–4 %. Such fat content was found in the LL and ST muscles in PL lambs slaughtered in both weight categories.

The values of the expressed juice for LL and ST muscles indicate that the meat of PW lambs has a better ability to hold its own water compared to PL sheep. The influence of the genotype on water-holding capacity (WHC) was also confirmed in the research by Sarı et al. (2019) conducted on Turkish sheep breeds. Also, Souza et al. (2016) found that meat from Santa Inez lambs had a lower WHC than Dorper crossbreeds. In turn, Cloete et al. (2012) did not confirm the influence of genotype on water-holding capacity in wool, dual-purpose and mutton sheep breeds.

## 4 Conclusions

The obtained results showed that the meat of lambs slaughtered at higher body weights was characterized by a larger diameter of muscle fibers. With increasing body weight at slaughter, an increase in the share of STO and FTO fibers and a decrease in the content of FTG fibers was observed in both tested muscles.

The ram lambs of the more primitive Polish Wrzosówka breed were characterized by a smaller diameter of all fiber types and a higher proportion of STO fibers compared to the meat-and-wool-purpose sheep.

The breed of lamb, muscle type and slaughter body weight had an impact on some meat quality characteristics.

Compared to Polish Lowland lambs, both investigated muscle types of Polish Wrzosówka were darker in color, had a greater saturation of redness and showed a lower content of intramuscular fat as well as a better value of expressed juice.

The breed of lamb, muscle type and body weight at slaughter had no effect on the shear force value, which is a measure of tenderness, one of the most important features in consumer evaluation of meat.

## Data Availability

The datasets used and analyzed during this study are available from the corresponding author on reasonable request.
